# Versatile format of minichaperone-based protein fusion system

**DOI:** 10.1038/s41598-019-51015-0

**Published:** 2019-10-21

**Authors:** Maria S. Yurkova, Olga A. Sharapova, Vladimir A. Zenin, Alexey N. Fedorov

**Affiliations:** 1 Bach Institute of Biochemistry, Research Center of Biotechnology of the Russian Academy of Sciences, 119071 Moscow, Russian Federation; 2 Tropogen Inc, Moscow, Russia; 3 Alder BioPharmaceuticals, Inc., 11804 N Creek Pkwy S, Bothell, WA 98011 USA

**Keywords:** Biochemistry, Biotechnology, Molecular biology

## Abstract

Hydrophobic recombinant proteins often tend to aggregate upon expression into inclusion bodies and are difficult to refold. Producing them in soluble forms constitutes a common bottleneck problem. A fusion system for production of insoluble hydrophobic proteins in soluble stable forms with thermophilic minichaperone, GroEL apical domain (GrAD) as a carrier, has recently been developed. To provide the utmost flexibility of the system for interactions between the carrier and various target protein moieties a strategy of making permutated protein variants by gene engineering has been applied: the original N- and C-termini of the minichaperone were linked together by a polypeptide linker and new N- and C-termini were made at desired parts of the protein surface. Two permutated GrAD forms were created and analyzed. Constructs of GrAD and both of its permutated forms fused with the initially insoluble N-terminal fragment of hepatitis C virus’ E2 protein were tested. Expressed fusions formed inclusion bodies. After denaturation, all fusions were completely renatured in stable soluble forms. A variety of permutated GrAD variants can be created. The versatile format of the system provides opportunities for choosing an optimal pair between particular target protein moiety and the best-suited original or specific permutated carrier.

## Introduction

Protein fusion systems are currently widely used for a variety of purposes, e.g. to improve expression yield and increase stability of passenger proteins, provide specific subcellular localization for a target, etc. A large number of carrier proteins have been used in fusion systems^1–5^. It has been shown that certain carriers are able to improve solubility of some target polypeptides. These carriers are small ubiquitin-like modifier (SUMO) protein^6^, maltose-binding protein^7–9^, immunoglobulin-binding domain of streptococcal protein G and several other small protein fragments^10,11^, highly charged proteins^12,13^, bacterial chaperones HSP70 and GroEL^14,15^. Co-expression of endoplasmic reticulum chaperones was also used to increase secretion level of the class II hydrophobin HFBI in *Pichia pastoris*^16^. The use of bacterial chaperones for stabilization of target proteins in different stages of biosynthetic production is reviewed previously in^17^. Still, with all the current progress and achievements in protein fusion technologies, there are many proteins of interest for research and biotechnology which are partly or completely insoluble by themselves and it would be beneficial to improve their solubility. Recently we developed a protein fusion system using a thermophilic minichaperone, GroEL apical domain, as a carrier^18^. The apical domain is a rather small 15 kDa polypeptide, has a monomer state, is able to bind various protein substrates, prevent their aggregation and promote folding ^19–22^. The basic idea for the system was to provide a protein carrier that may efficiently bind different hydrophobic polypeptides and thus alleviate their poor solubility patterns. Indeed, the minichaperone-based fusion system has shown great capacity in drastically enhancing solubility and stability of two unrelated insoluble passenger inserts^18^.

Interactions of a carrier with various substrate polypeptides almost certainly require specific mutual orientation of the two protein moieties in a fusion polypeptide, which may be difficult to achieve in a single polypeptide chain. In this work, the goal was to develop an approach for creating a versatile carrier that will accommodate, i.e. will be able to efficiently bind, various “difficult” proteins. Making protein permutations has been chosen as a strategy with regard to these goals.

The approach of creating protein permutations relies on the fact that in many proteins N- and C-termini are located in close proximity to each other^23–25^. These termini can be linked together and new termini recreated either by chemical treatments or by means of gene rearrangements. Two elegant works have laid experimental foundation in this area. Circular and circular permutated protein variants were made with bovine pancreatic trypsin inhibitor as a framework^26^. The circular protein was obtained by chemically ligating the natural termini; the circular permutated form was further made by recreating termini at new positions by proteolysis at a specific amino acid residue. A genetic approach for making circular permutated proteins by rearranging the order of DNA fragments in a gene encoding the target protein has been developed in the pioneering work by K. Luger and colleagues^27^. This approach has been applied to many proteins and is used for various purposes. For one, it has been used to address a question in protein folding of whether the natural termini and the given order of polypeptide chain segments are critical to the folding pathway, stability and final structure of proteins^27–31^. It’s been shown that circular permutations may affect various properties of proteins often improving some desired characteristics. Positive effects on thermostability^32^ and catalytic activity^33^, lesser proteolytic susceptibility^34^, altered substrate binding and specificity^33,35^ have been reported. Thus, making circular permutations has proven to be a very potent tool in protein engineering.

## Results and Discussion

### Design of GroEL apical domain permutated constructs convenient for use as carriers for target proteins

**Figure 1:**
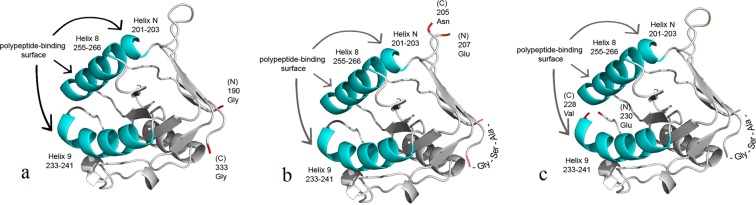
Three-dimensional structures of *T. thermophilus* apical domain and its two permutations. a – original GrAD, b – permutated GrAD207, c – permutated GrAD230. To illustrate the positions of newly created N- and C-termini the published structure of *T. thermophilus* GroEL apical domain available in pdb-bank at http://www.rcsb.org/structure/1SRV was used. For manipulations with the structure perspective the program PyMOL2.3 was used. Original or newly created N- and C-termini are shown in red, alpha helices 8, 9 and N, forming polypeptide- binding surface, shown in turquoise. The Ala-Ser-Gly linker that fuses the original termini of the polypeptide chain is shown in b and c.

The GrAD is structurally isolated from the rest of GroEL, has a relatively small size of about 15 kDa and its recombinant form exists as a monomer. Most importantly from a functional viewpoint, GrAD retains the ability to efficiently bind different substrate proteins. We have demonstrated that GrAD used as a carrier for insoluble proteins is able to maintain passenger inserts in soluble functional form^18^. We used *T. thermophilus* GrAD from a thermophilic source to take additional advantage of its thermostability. For the purpose of convenient chemical release of a passenger polypeptide, Met-less GrAD was constructed and used thereafter. In this study, we have developed a strategy in order to make GrAD-based versatile carriers suitable for diverse passenger proteins. Our approach for making GrAD a truly versatile carrier able to bind diverse passenger proteins was making permutations, i.e. GrAD polypeptide chain constructs with fused original termini and newly created N- and C-ends. Indeed, interactions of substrate proteins with GrAD are individual, they may require mutual orientation of the two protein moieties that is impossible or difficult to achieve upon linking a passenger protein to a GrAD polypeptide terminus at its original location, but may be readily achieved at an alternative GrAD terminus location. Consequently, fusion of a target protein to natural N- or C-termini of GrAD may be insufficient with regard to its stabilization in a soluble form for steric reasons. Placing target proteins at specific parts of permutated GrAD may solve this problem. It is clearly seen that Gly190, the N-terminal residue of the original GrAD construct, and Gly333, its C-end, are located in close proximity (see Fig. 1a).

The linker of three amino acid residues, Ala-Ser-Gly, was introduced to fuse the termini of the polypeptide chain. Two alternative positions for new termini have been chosen. The first position for the new termini has been placed in the unstructured flexible loop after the helix N of the original polypeptide chain. The N-terminus has been made at Glu 207 and the C-terminus at Asn 205 (see Fig. 1b; this permutation was termed GrAD207).

The new termini of the second permutation precede helix 8 in the polypeptide chain and in the three-dimensional structure reside between helices 8 and 9. These helices form major surface recognizing substrate polypeptides^36^. The new N-terminus has become Glu 230 and the C-terminus – Val 228 (see Fig. 1c; this permutation was termed GrAD230). Thus, these new termini became immediately adjacent to the polypeptide-binding surface of the domain.

**Figure 2:**
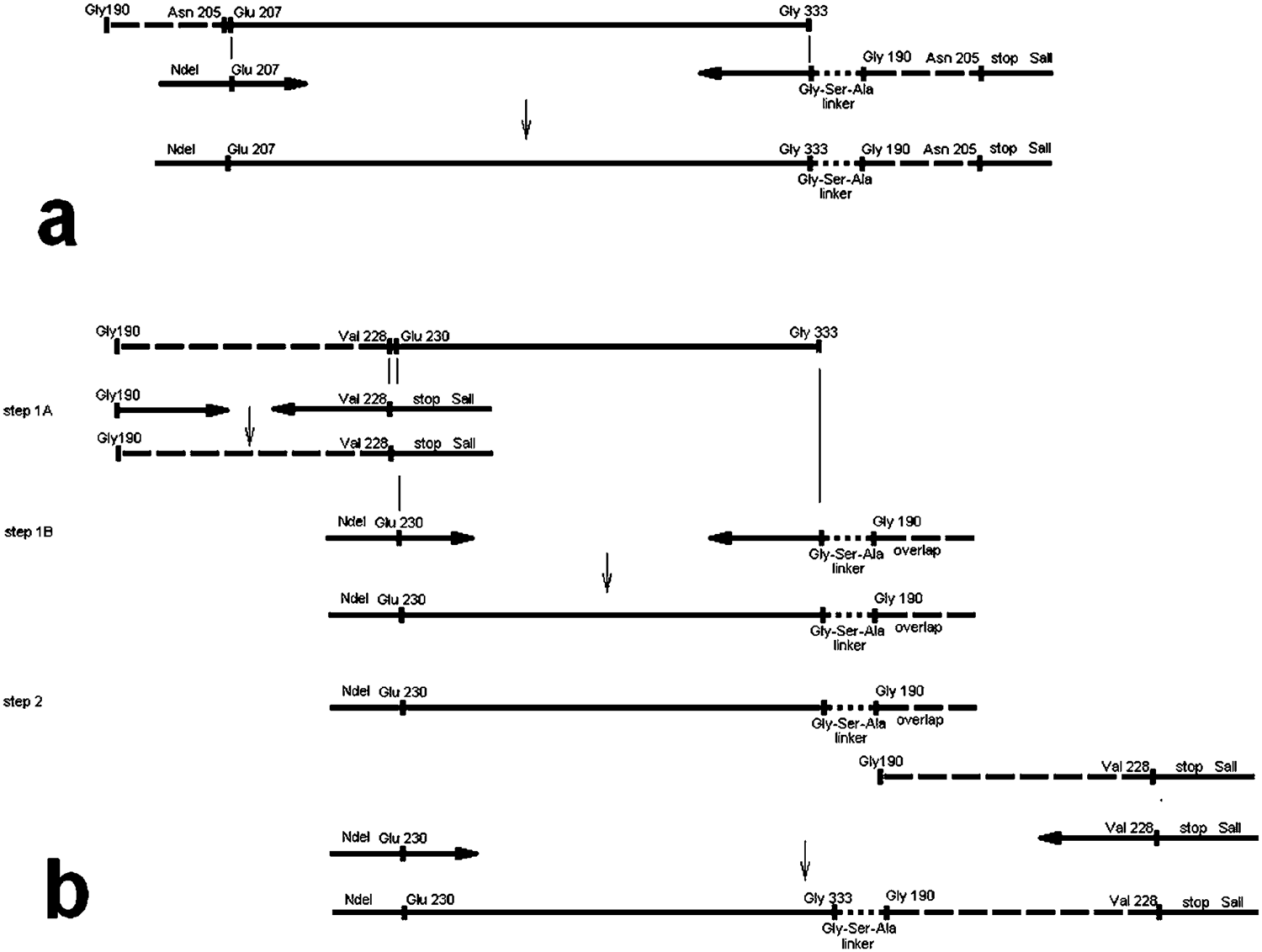
Diagram of making gene constructs for GrAD permutations; a – GrAD207, b – GrAD230.

The schemes of making corresponding gene constructs are presented in Fig. 2a,b. The GrAD207 gene construct was obtained by a single-round amplification of the original template (Fig. 2a). The upstream amplification primer provided the codon for the initiator Met within the NdeI restriction site followed by GrAD nucleotide sequence starting with Glu 207 codon. The downstream primer after the GrAD specific sequence which ends with the Gly 333 codon was extended to encode a linker to join original N- and C- termini and the sequence of the 5′ end of the original gene from Gly 190 codon to Asn 205 codon. This gene construct was cloned further by standard procedures (see Methods).

For the GrAD230 gene construct, the two segments of the desired gene were separately amplified (steps 1a and 1b in Fig. 2b). Amplification of the new 5′ fragment of the gene construct was performed with the upstream primer providing the initiator Met codon and GrAD sequence starting at the Glu 230 codon. The downstream primer included a 3′ end GrAD sequence, a sequence encoding a linker to join original N- and C- termini and the sequence of the 5′ end of the original gene template. Amplification of the new 3′ segment was performed to provide the gene fragment from the original 5′ end of the GrAD-encoding gene to the position of the new 3′ end of the permutation ending with an added stop codon. Then, the two segments were amplified together to make a new complete sequence of the permutated form of GrAD (step 2, Fig. 2b) and this gene construct was also cloned by standard procedures (for details, see Methods).

**Figure 3:**
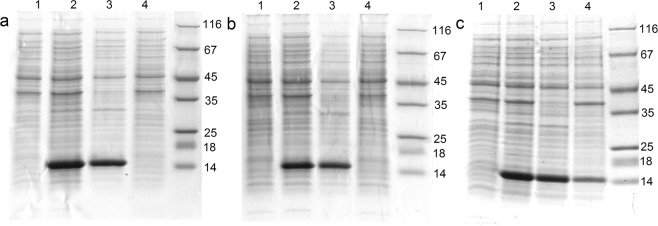
Expression and distribution of permutations of Met-less *T. termophilus* apical domain; a – GrAD, b – GrAD207, c – GrAD230. For all panels: line 1 – before induction, line 2 – after induction; line 3 – soluble cell proteins; line 4 – insoluble cell proteins; molecular mass standards for all figures are 14, 18, 25, 35, 45, 67, and 116 kDa.

Both permutated forms of GrAD were expressed and analyzed for basic properties. As shown in Fig. 3b for GrAD207 and Fig. 3c for GrAD230, lines 1 and 2, the permutations were expressed at high yields mostly in a soluble form. Lanes 3 in all panels of this figure represents soluble cell proteins and lanes 4 show insoluble pellets in normalized proportions for direct comparison of these fractions. Figure 3a shows the expression and distribution of non-permutated GrAD for comparison.

**Figure 4:**
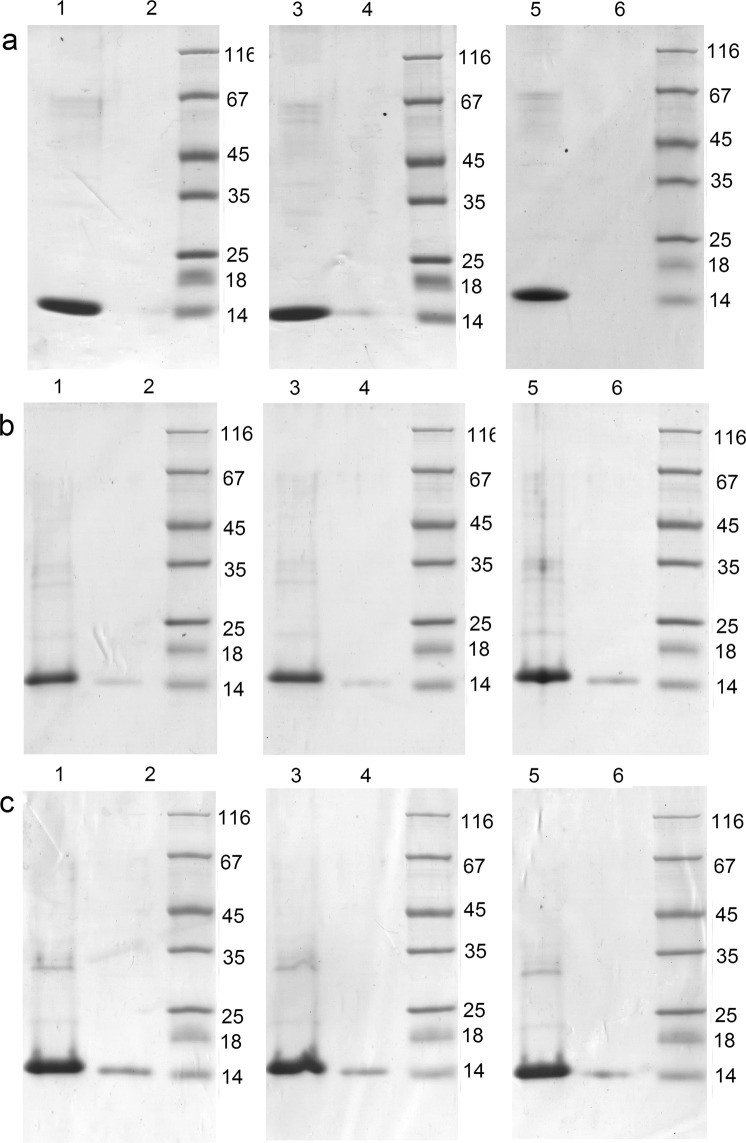
Properties of permutations of Met-less *T. termophilus* apical domain; a – GrAD, b – GrAD207, c – GrAD230. For all panels: lines 1 and 2 – supernatant and pellet after heating the proteins at 62 °C for 5 min; lines 3 and 4 – supernatant and pellet after freezing-thawing; lines 5 and 6 – supernatant and pellet after lyophilization-re-dissolving.

Soluble proteins, GrAD and its permutations, were purified by ion-exchange chromatography, as described in Methods. The comparison of their properties was conducted with purified proteins. In Fig. 4 lines 1 and 2 for all panels represent their thermostability.

The GrAD207 fully retained thermostability upon exposure to an elevated temperature 62 °C for 5 min (Fig. 4b lane 1 vs lane 2) as did the initial GrAD (Fig. 4a lane 1 vs lane 2). The GrAD230 (Fig. 4c lane 1 vs lane 2) tended to partially aggregate after heating, over 70% retained solubility after the procedure. An important issue is, of course, the ability of permutated GrAD to bind proteins. Both obtained permutations were able to bind to resin-coupled recombinant E2 protein of hepatitis C virus, a highly hydrophobic insoluble protein^37^ (data not shown). Thus, it can be concluded that the two permutated GrAD versions maintain solubility of the original minichaperone, while differ in thermostability, and retain the ability to bind hydrophobic substrate polypeptides.

The permutated forms were also subjected to freezing-thawing and lyophilization-re-dissolving, to compare their stability in commonly used biochemical manipulations. The results are shown in Fig. 4 b,c, lines 3–6 for each panel. GrAD207 (Fig. 4b) demonstrated the same stability as GrAD (Fig. 4a), while GrAD230 (Fig. 4c) was somewhat more labile, but still retaining about 90% of the protein in soluble state.

As a next step, relatively long flexible linkers at the C-termini of permutated GrAD constructs were introduced (see Methods) upon making fusion constructs to better accommodate passenger proteins for proper interaction with the substrate-binding surface of GrAD. The sequence encoding recognition site for enterokinase was included in the linkers to have an option to cleave a passenger insert from the carrier.

### Permutated GrAD constructs fused to insoluble target via corresponding recreated C-termini

The permutated GrAD forms were used to make fusions with the N-terminal fragment of the E2 structural glycoprotein of hepatitis C virus, a 45 aa hydrophobic insoluble polypeptide, as was done with the original GrAD^18^. For comparison, we used fusions of the same polypeptide with GrAD, E2 fused either with N-terminus or C-terminus of GrAD.

**Figure 5:**
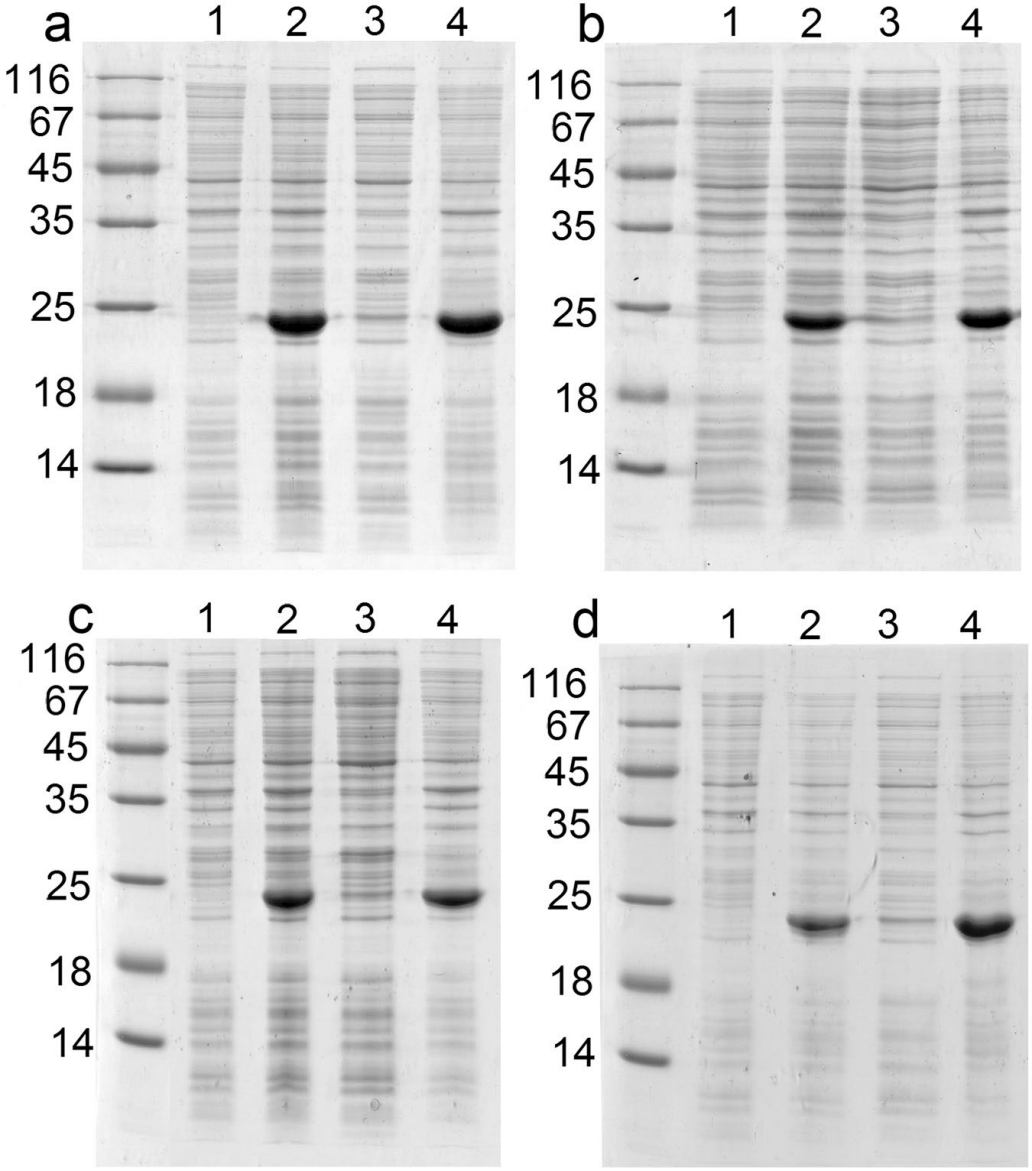
Expression and distribution of fusions of GrAD and its permutated forms with N-terminal fragment of E2: a – E2-GrAD, b – GrAD-E2, c – GrAD207-E2, d – GrAD230-E2. For all panels: line 1 – before induction, line 2 – after expression; line 3 – soluble cell proteins; line 4 – pellet after centrifugation of lysed cells at 13000 g for 15 min.

Expression and properties of fusions of N-terminal fragment of HCV E2 protein with two permutated GrAD constructs via their corresponding C-ends were analyzed and compared with non-permutated GrAD fusions. Upon expression *in vivo* at 37 °C, all of the fusions formed inclusion bodies (Fig. 5).

In the previous study, we tested different temperature conditions to see if fusions of GrAD with the N-terminal fragment of HCV E2 protein and with HPV type 16 E6 protein, which is also insoluble if unassisted, can be obtained in soluble forms upon expression^18^. They were partially soluble upon expression at a lower temperature, but we had chosen inclusion bodies for further work, and, thus, did not endeavour to express fusions with permutated GrAD in soluble forms in this work. For further experiments, fusion proteins obtained from the inclusion bodies were used as starting material. The thoroughly washed inclusion bodies contained substantially purified target fusions. The material was dissolved in 8 M urea, purified by cation-exchange chromatography and then allowed to refold by dilution in appropriate native buffers. The yield of refolding for all the constructs was no less than 90%. Renatured proteins stayed predominantly in a soluble fraction. They were further purified and concentrated by an additional chromatography step and then used for analysis.

**Figure 6:**
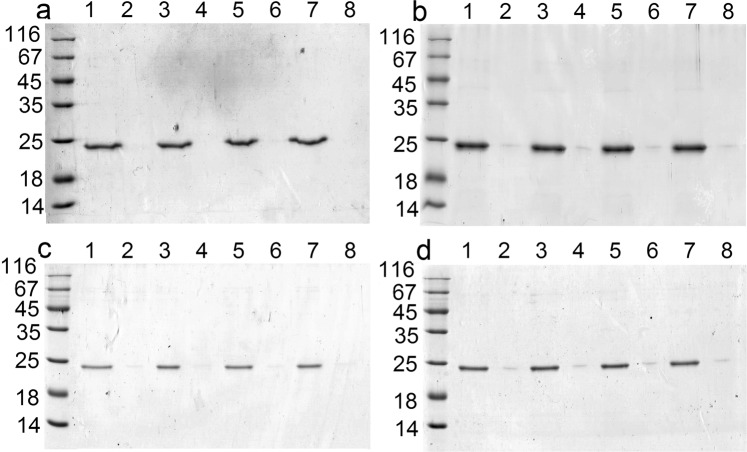
Distribution of renatured GrAD constructs after storage at + 4 °C; a – E2-GrAD, b – GrAD-E2, c – GrAD207-E2, d – GrAD230-E2. For all panels: line 1, 3, 5, 7 – soluble proteins, line 2, 4, 6, 8 – insoluble proteins after 0, 3, 7, 10 days of storage at 4 °C.

Purified concentrated proteins were kept at +4°C, aliquots for further analysis were taken immediately after concentration and after 3, 7 and 10 days of storage. Soluble and aggregated forms were separated by centrifugation and analyzed by SDS-PAGE. All obtained fusions were soluble and retained solubility at tested high concentrations for the entire duration of the tests (see Fig. 6).

**Figure 7:**
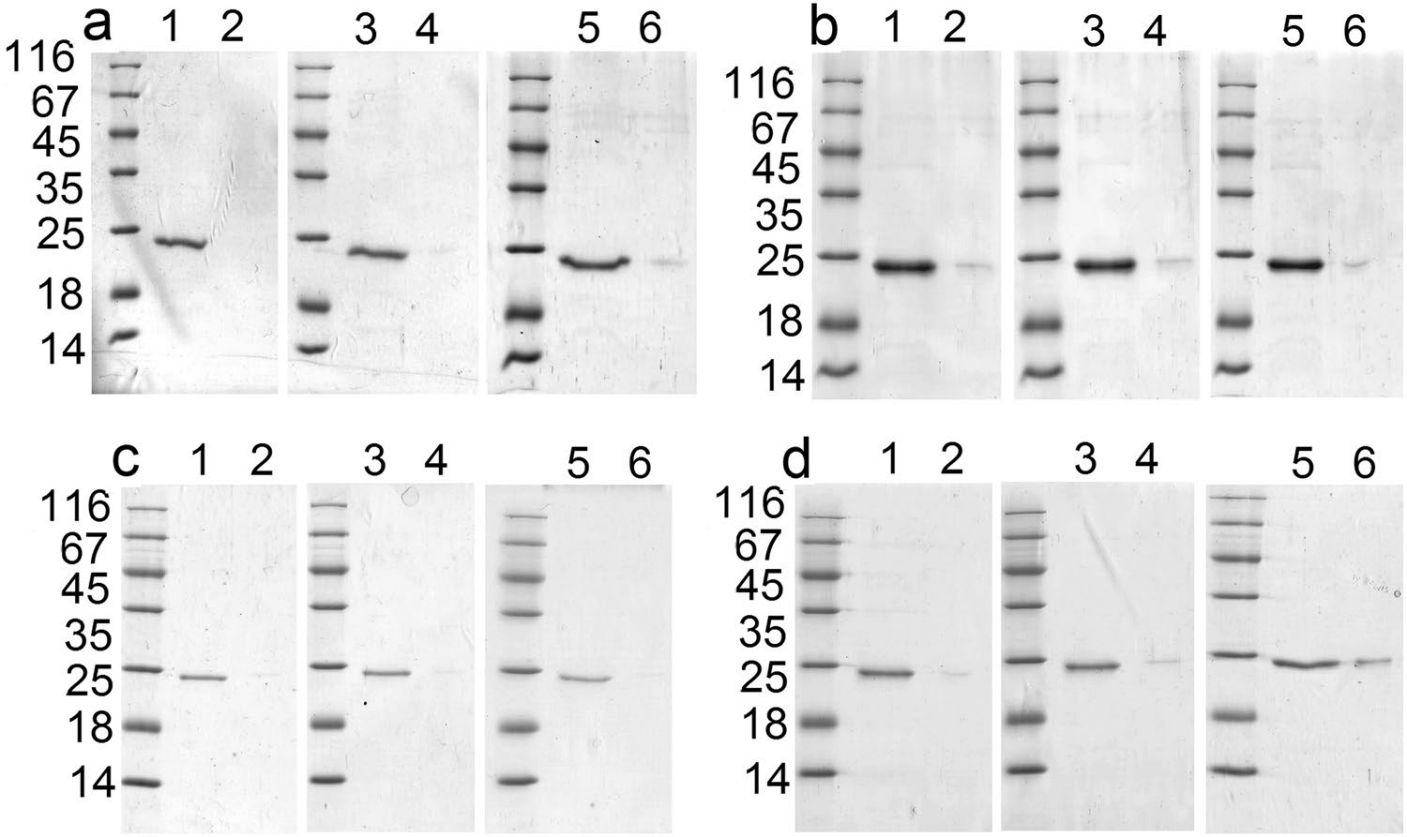
Distribution of permutated GrAD constructs after heating, freezing-thawing and lyophilization-re-dissolving procedures: a – E2-GrAD, b – GrAD-E2, c – GrAD207-E2, d – GrAD230-E2. For all panels: line 1 – supernatant, line 2 – pellet after heating at 62 °C for 5 min; line 3 – supernatant, line 4 – pellet after freezing-thawing; line 5 – supernatant, line 6 – pellet after lyophilization-re-dissolving.

The fusions with permutated GrAD forms were tested for thermostability and subjected to freezing-thawing and lyophilization-re-dissolving procedures. These results are shown in Fig. 7. GrAD207-E2 construct (Fig. 7c) demonstrates the same high stability, as the constructs with GrAD, E2-GrAD and GrAD-E2 (Fig. 7a,b). GrAD230-E2 construct, while wholly stable at storage (Fig. 6d), demonstrated some lability after biochemical manipulations (Fig. 7d), like the carrier GrAD230 itself (Fig. 4c), but still the vast majority of the protein remained soluble after freezing-thawing and lyophilization-re-dissolving procedures.

The results indicate that, similarly to the initial GrAD-based fusions, aggregation upon expression *in vivo* is not an intrinsic property of these permutated minichaperone-based fusions, but is a result of a biosynthetic folding pathway under the conditions of their overexpression. Protein aggregation *in vivo* has been studied in some detail^38–40^. Protein folding in a cell is a complex process, which may start early upon polypeptide biosynthesis^41–43^ and for many proteins require involvement of specific chaperones or their networks^43–46^. Refolding *in vitro* for many proteins may proceed spontaneously with or without the participation of chaperones.

In conclusion, we have successfully applied a permutation strategy to GroEL apical domain. It is known from the analysis of different permutations of a large number of proteins that linking natural termini and recreating the new ones may have all the magnitude of potential effects on structure, stability, folding patterns, etc^23–25^. Most importantly, it is possible to get permutated versions with untouched basic structural parameters of the original protein and in certain cases even to improve some of them. Both permutations described in this article were designed for further accommodation of passenger inserts in fusion constructs. Of the two permutations, one, GrAD207, retained basic patterns of the original counterpart, and the second, GrAD230, was entirely soluble but less thermostable. Clearly, other permutations can be made and tried for their patterns and suitability as carriers for the fusion system. While outside the scope of this study, it would be interesting to perform a systematic analysis of the minichaperone permutations and examine effects of termini positions on their structural characteristics and functionality with regard to binding hydrophobic protein substrates, their refolding etc. For the basic goals of this study, both permutated GrAD versions were substantially similar to its original counterpart and could have been used as carriers in the fusion system. Indeed, both permutated GrAD-based fusion constructs with the originally insoluble passenger insert have shown, after renaturation, high solubility, stability and retained solubility after freezing and lyophilization. In other words, permutated GrAD forms may be successfully used in fusion constructs with “difficult”, prone to aggregation polypeptides to produce their soluble stable forms. Whatever the specific mechanisms of improved solubility of poorly soluble passenger inserts within the original and permutated minichaperone-based fusions may be, basically it should be a result of shielding of some segments of the inserted polypeptide from bulk solution as it is described in detail for chaperone binding to substrate proteins^44,46,47^. It has been demonstrated that the minichaperone is able to mediate folding of certain proteins^21,48–50^. The minichaperone does not possess ATPase activity^49^ and, interestingly enough, in certain cases the entire GroEL may also execute its folding functions without ATP^51^. Minichaperone-based carriers may also mediate folding for fused proteins in the same manner, allowing them to retain at least some functional characteristics within the fusion constructs. In the experiments presented in this study, there were no substantial differences in patterns of the fusion constructs of the original form of the carrier and its two permutated variants. However, it is clear from general considerations that a capability to place target proteins at different parts of the carrier in order to realize optimal mutual orientation(s) of the two protein moieties can make the system truly generally applicable. Chaperones have become subjects of protein engineering^52^. The full-length GroEL particle engineered by directed evolution approaches can acquire new substrate protein preferences^53,54^, it can be fused into the single polypeptide chain to cage a target protein^15^. Stability of the *E. coli* minichaperone can be substantially improved by structure-driven amino acid substitutions^20^.

Further analysis of the importance of particular N- and C-termini for minichaperone stability, other physico-chemical properties and functionality with regard to binding substrate polypeptides and their folding may provide additional insight into chaperone functioning and activities. Also, the described approach for making permutated minichaperone forms show, we believe, that minichaperone patterns can be engineered to the needs of specific target proteins in the developed fusion system.

## Methods

### Gene and plasmid construction

pGrAD construction is described in^18^.

#### pGrAD230

During the first step, the two segments of the desired gene construct were separately amplified (steps 1a and 1b in Fig. 2b) using *T. thermophilus* GroEL apical domain (GrAD) sequence^18^ as a template. Step 1a was performed using forward primer F1: 5′-GGGTACCAGTTTGACAAGGGGTAC-3′ and reverse primer R1: 5′-ACGCATCGGTCGAC**TTA**GACGTTGGAGACCTTCTTCTCCACG-3′. It produced gene fragment encoding amino acid residues from 190 to 228 of the full length GroEL; the recognition site for SalI is underlined, the stop codon is in bold. Step 1b was performed using forward primer F2: 5′-GGGAATTCCATATGGAGCTCCTCCCCATCCTGGAG-3′ and reverse primer R2: 5′-GTACCCCTTGTCAAACTGGTACCC**CGCGCTACC**GCCGCCCACGATGGTGGT-3′. This gene fragment contained an initiator ATG incorporated into an NdeI site (underlined), the apical domain sequence encoding amino acid residues 230 to 333, followed by a linker sequence encoding Gly Ser Ala (in bold) and, finally, the apical domain sequence encoding amino acid residues 190 to 196 to provide an overlap for the next combined amplification. Combined amplification using purified fragments obtained at steps 1a and 1b as templates and two primers, F2 and R1 (step 2 in Fig. 2b) produced a permutated nucleotide sequence (GrAD230). This fragment was digested with SalI and NdeI restriction enzymes and cloned into modified pET11c yielding expression vector pGrAD230.

#### pGrAD207

 The GrAD sequence was first amplified using two oligonucleotides, forward primer F3: 5′-GGGAATTCCATATGGAGACGCTGGAAGCGGTCCTC-3′ and reverse primer R2 (see above), underlined is site for NdeI. It provided GrAD sequence encoding amino acid residues 207 to 333, followed by a linker sequence encoding Gly Ser Ala and, finally, the sequence encoding amino acid residues 190 to 196. The obtained gene fragment was subjected to amplification with the same forward primer F3 and reverse primer R3: 5′-ACGCATCGGTCGAC**TTA**GTTGGTGACGAAGTAGGGGGAGATGTACCCCTTGTCAAACTGGTACCC-3′, which added specific GrAD sequence to aa residue 205 followed by a stop codon (shown in bold) and a recognition site for SalI (underlined) (Fig. 2a). These amplifications produced the GrAD207 nucleotide sequence. This fragment was cloned into a modified pET11c yielding expression vector pGrAD207.

### Extended GrAD constructs used as fusion carriers with linkers to their 3′ ends

The construction of extended GrAD is described in^18^. The same approach was used for GrAD207 and GrAD230. Two separate PCR amplifications were performed using either pGrAD230 or pGrAD207 as templates with following pairs of primers, F2 and R5: 5′-CTTGTCATCGTCATCGCCGGCACCAGAACCAGAGACGTTGGAGACCTTCTTCTCCACG-3′ (for pGrAD230), F3 and R6: 5′-CTTGTCATCGTCATCGCCGGCACCAGAACCAGAGTTGGTGACGAAGTAGGGGGAGATGTACCCC-3′ (for pGrAD207). Underlined is the NaeI recognition site. Amplifications produced extended nucleotide sequences of GrAD permutations (GrAD230-link and GrAD207-link) for fusion with passenger inserts. Both of them contained 3′ end extensions encoding aa sequence SGSGAGDDDDK downstream corresponding permutated GrAD sequences.

### Fusions of E2 N-terminal fragment to the 3′ ends of extended GrAD constructs

First, PCR amplification was performed using E2 glycoprotein sequence of hepatitis C virus genotype 1b as a template^37^ and two primers, forward primer E2f1: 5′-*GCCGGCGATGACGATGACAAG*ATCCAGCTTGTGAATACCAACGGC-3′ and reverse primer E2r1: 5′-ACGCATCGGTCGAC**TTA**GCGCTCCGGGCACCC-3′. It produced nucleotide sequence encoding aa residues 404 to 448 of HCV polypeptide (the N-terminal fragment of E2 protein), site for SalI (underlined), stop codon (bold characters) and sequence (in italic) for fusing with extended GrAD permutations. The combined PCR amplifications were performed with extended permutated GrAD gene fragments and E2 gene fragment using specific forward primers for corresponding GrAD fragments and reverse primer E2r1. The obtained fusion gene constructs were cloned into modified pET11c.

All obtained constructs were confirmed by DNA sequencing analysis.

### Fusion of E2 N-terminal fragment to the 5′ end of GrAD

PCR amplification was performed using E2 glycoprotein sequence of HCV genotype 1b as a template and two primers, forward primer E2f2: 5′-GGAGATTCCATATGATCCAGCTTGTGAATACCAACGGC-3′ and reverse primer E2r2: 5′-CTTGTCATCGTCATCGCCGGCACCAGAACCAGAGCGCTCCGGGCACCC-3′. It produced E2-link nucleotide sequence encoding residues 404–448 of virus polypeptide (the N-terminal fragment of E2 protein) with the 3′ end extension encoding aa sequence SGSGAGDDDDK. Recognition sites for NdeI (in E2f2) and NaeI (in E2r2) are underlined. PCR amplification using pGrAD as a template and two primers, forward primer F4: 5′-GCCGGCGATGACGATGACAAGGGGTACCAGTTTGACAAGGGGTAC-3′ and reverse primer ADr 5-ACGCATCGGTCGACTTAGCCGCCCACGATGGTGGT-3′ produced link-GrAD nucleotide sequence encoding GrAD with 5′ end extension essential for fusion with E2-link (underlined). Combined PCR amplification using E2-link and link-GrAD as templates with primers E2f2 and ADr produced nucleotide sequence encoding E2-GrAD fusion construct. This construct was cloned into pET11c yielding expression vector pE2-GrAD.

### Protein production

All proteins were expressed in *E. coli* BL21(DE3) grown in LB media at 37 °C. Expression was induced by adding isopropyl-β-D-thiogalactopyranoside to a final concentration 0.4 mM at a density of 0.4 OD600. Cells were harvested by centrifugation 3 hours after induction.

### Protein purification

GrAD, GrAD230 and GrAD207 purification and assays were basically conducted as described in^18^ for GrAD. The cell pellet was resuspended in PBS (KH_2_PO_4_ 1.7 mM, Na_2_HPO_4_ 5.2 mM, NaCl 150 mM) pH 7.4 with 0.1 M NaCl and 1 mM 4-(2-aminoethyl)benzenesulphonyl fluoride (AEBSF) at room temperature (RT). The suspension was sonicated at 0 °C and centrifuged. The supernatant was incubated for 10 min at 62 °C and centrifuged again. Urea was added to 8 M final concentration to the supernatant and it was incubated for at least 1 h at RT. The supernatant was dialyzed against 10 mM Tris, 8 M urea, pH 8.0 to NaCl final concentration 10 mM and final pH 8.0. This solution was loaded onto Q Sepharose column (GE Healthcare) equilibrated in the same buffer. Then, the unbound material and urea were washed out. The protein was eluted by NaCl gradient and dialyzed against PBS.

### Purification of fusion proteins

The cell pellet was resuspended in PBS, pH 7.4 with 0.1 M NaCl and 1 mM AEBSF at RT. The suspension was sonicated at 0 °C and centrifuged. The pellet was resuspended in PBS pH 7.4 with 0.1 M NaCl and 0.1% sodium deoxycholate, sonicated and centrifuged. The pellet was then twice washed in PBS as described above. Purified inclusion bodies (IB) were resuspended in solubilization buffer (10 mM KH_2_PO_4_ pH 6.5, 8 M urea and 1 mM DTT), incubated overnight and centrifuged. This solution was loaded onto an SP sepharose column (GE Healthcare). In these conditions, most of the cell proteins bound to the column, while GrAD fusion proteins did not. The unbound material was collected and, in the case of GrAD fusions (E2-GrAD and GrAD-E2), renatured by means of drop-wise dilution upon fast mixing in 100X excess of renaturation buffer (20 mM tris pH 8.0 with 20 mM NaCl) to final protein concentration of about 0.01–0.02 mg/ml, incubated overnight at 4 °C with constant stirring and centrifuged. In the case of fusions with permutated forms of GrAD, GrAD207 and GrAD230, the unbound material after SP Sepharose chromatography was renatured by dialysis against 10 mM potassium phosphate buffer pH 7.0. Renatured proteins were loaded on Sepharose Q column, eluted in the gradient of NaCl and dialyzed against PBS.

### Thermostability assay

Samples of purified proteins at concentrations 0.1–0.5 mg/ml were heated at 62 °C for 5 min in a heater unit (VWR). After that, samples were centrifuged to separate soluble fraction from precipitated material. Aliquots of soluble fraction and precipitated material, normalized to the same amount of the initial protein sample, were analyzed by Tris-Tricin PAGE^55^ under reducing conditions for GrAD and its permutated forms, or by PAGE under reducing conditions for the constructs with N-terminal fragment of E2.

### Solubility assay

Samples of soluble renatured proteins were concentrated at Amicon concentration unit (Millipore) to final protein concentration 1–2 mg/ml. Aliquots for analysis were centrifuged to separate soluble fraction from precipitated material. Aliquots of soluble fraction and precipitated material, normalized to the same amount of the initial protein sample, were analyzed by Tris-Tricin PAGE^55^ under reducing conditions for GrAD and its permutated forms, or by PAGE under reducing conditions for the constructs with N-terminal fragment of E2. For further analysis, purified concentrated proteins were used.

### Stability assays

for all proteins were conducted as described in^18^. Aliquots for the analysis of protein stability in soluble form were taken immediately after concentration and 1, 3 and 5 days after storage at +4 °C. For freezing-thawing assay, aliquots of proteins were kept frozen at least overnight at −20 °C, then thawed. Lyophilization was performed with frozen protein solutions in PBS buffer at concentrations ~ 1–2 mg/ml. Dried material was re-dissolved by adding an initial amount of de-ionized water. Distribution of proteins between a soluble fraction and precipitated material was analyzed as above.

## Data Availability

All data generated or analysed during this study are included in this published article.
